# Phenotypic Alteration of an Established Human Airway Cell Line by Media Selection

**DOI:** 10.3390/ijms24021246

**Published:** 2023-01-08

**Authors:** Galit Livnat, Jessica D. Meeker, Alicia J. Ostmann, Lauren M. Strecker, John P. Clancy, John J. Brewington

**Affiliations:** 1 Pediatric Pulmonology and CF Center, Carmel Medical Center, Haifa 3100000, Israel; 2 Ruth and Bruce Rappaport Faculty of Medicine, Technion-Israel Institute of Technology, Haifa 3109601, Israel; 3 Department of Pediatrics, College of Medicine, University of Cincinnati, Cincinnati, OH 45299, USA; 4 Division of Pulmonary Medicine, Cincinnati Children’s Hospital Medical Center, Cincinnati, OH 45299, USA; 5 Cystic Fibrosis Foundation, Bethesda, MD 20810, USA

**Keywords:** cystic fibrosis, CFTR, tissue culture, growth media, CFBE41o-

## Abstract

Cystic Fibrosis (CF) is caused by mutations in the CF transmembrane conductance regulator (CFTR), a chloride/bicarbonate channel. Many studies utilize human airway cell models (cell lines and primary cells) to study different aspects of CFTR biology. Media selection can alter the growth and differentiation of primary cells, yet the impact on stable airway cell lines is unclear. To determine the impact of media and growth conditions on CFBE41o- cells stably transduced with wild-type or F508del CFTR, we examined four commonly used growth media, measuring epithelial and mesenchymal markers, as well as CFTR expression, maturation, and function. The selection of growth media altered the expression of epithelial and mesenchymal markers in the cell lines, and significantly impacted CFTR expression and subsequent function. These results highlight the importance of media selection to CFTR and cell line behavior and should be considered in both studies of primary human airway cells and stable cell lines.

## 1. Introduction

Cystic Fibrosis (CF) is an autosomal recessive disease affecting over 100,000 people worldwide [1]. CF is caused by the loss of function of the Cystic Fibrosis Transmembrane Conductance Regulator (CFTR) protein, a member of the ATP-binding cassette family, which functions as a chloride and bicarbonate channel in the apical membrane of multiple epithelia [2]. Both human airway cell lines and primary cells are established models for CFTR-based studies, including epithelial biology and the development of CFTR-based therapies [3,4,5]. One frequently used cell line is the CFBE41o- line, originally described by Gruenert and colleagues in 1993 [6]. These cells constitute an SV40-immortalized human airway epithelial cell line derived from an individual with cystic fibrosis. CFBE41o- cells express several key epithelial markers, though they do not polarize into a classical pseudo-columnar epithelium [6]. Over time, this line has been stably transduced with wild-type (wt) or F508del *CFTR* cDNA under the control of a CMV promoter, thus allowing for high amounts of CFTR protein expression. These cells are often used to study CFTR due to their ease of growth, stable behavior over time, and their epithelial origin [4,7]. Critically, this CFBE41o- line has frequently been utilized to quantify the rescue of abnormal CFTR as part of a body of work leading to the currently available CFTR modulators as well as the general understanding of CFTR biology. The overexpression of wild-type and mutant CFTR allows for the improved isolation of CFTR protein by Western blot with the quantification of both the immature B band and the fully glycosylated C band [8,9,10]. This same overexpression generates a strong CFTR functional signal in well-validated assays such as short-circuit current measurement and halide or chloride efflux [9,11,12,13]. As such, this cell line has been a mainstay of CFTR-focused studies over the years [14,15,16]. 

Unlike primary airway cells, many cell lines are grown in media that were not developed to support epithelial differentiation. For example, CFBE41o- cells were originally described in culture with Minimal Essential Media (MEM) supplemented with serum, which is among the most widely used strategies for cell line culture [6]. Over time, several studies have employed other growth media with this cell line, including supplements designed to encourage epithelial differentiation, while some studies have not explicitly reported the growth media used for the respective cell culture [17,18,19]. This is of particular relevance to human airway epithelia, in which several specialized media have been developed and used to support seminal studies of CF airway epithelial behavior, including mucus dehydration, microbial killing, and, ultimately, CFTR modulator development [20,21,22]. Recent studies have demonstrated notable changes in primary cell physiology when cultured in media with different additives [23]. Differences in the physiological behavior of the CFBE41o- line have been reported when the cultures are grown in submerged versus air-exposed conditions [24]. To our knowledge, however, no studies have examined how the CFBE41o- cell line’s behavior and physiology are impacted by media selection. 

In the current report, we examined the wt and F508del *CFTR* cell lines’ behavior under four different growth conditions, including three specifically developed to support primary human airway epithelia growth and differentiation. The results confirm the importance of media selection when applied to stable cell lines expressing wt or F508del *CFTR* and should be considered when designing studies of CFTR and epithelial biology.

## 2. Results

### 2.1. Markers of Epithelial and Mesenchymal Phenotypes

To assess the effect of media selection on the phenotypic characteristics of this bronchial epithelial cell line, CFBE41o- cells of the same passage (all less than passage 30) were plated onto semi-permeable inserts and grown for 10 days in different growth media. Commonly utilized media for airway epithelial biology studies were selected, including MEM [25], an Ultroser-G^TM^-based media (USG) [26], a media specifically optimized for air–liquid interface culture (ALI media) [27], and a commercially available proprietary media (PneumaCult^TM^—ALI media) (PC media) [28]. The cells were lysed, and protein and RNA were isolated. 

**Figure 1:**
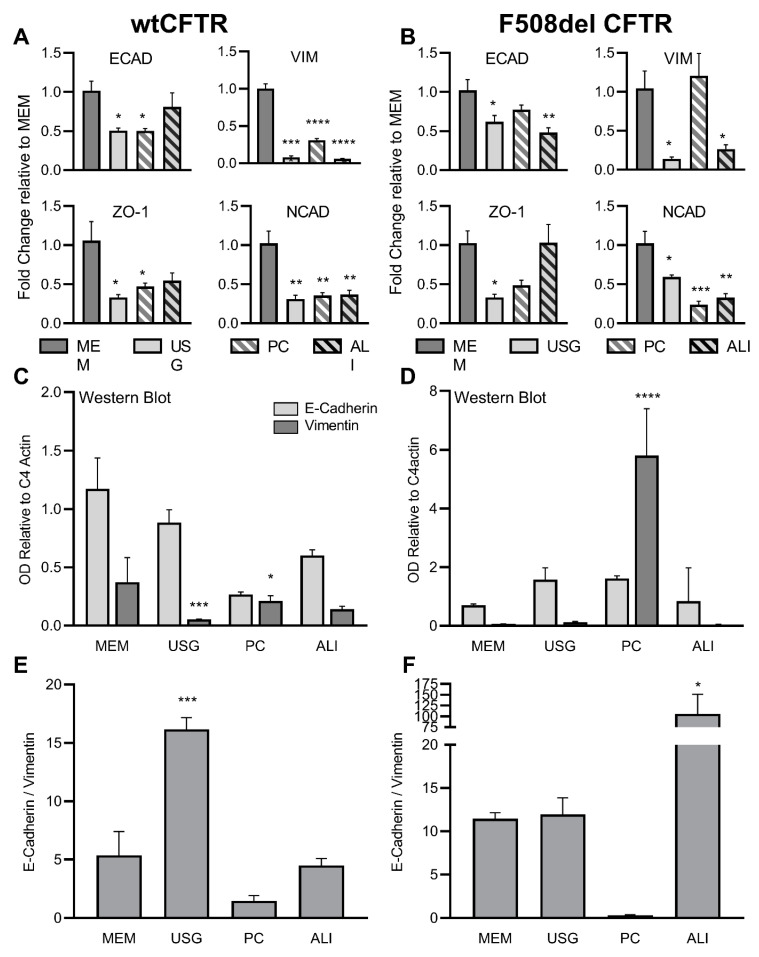
Media selection impacts epithelial and mesenchymal marker expression in wtCFTR and F508del CFBE41o- cells. RT qPCR was performed for e-cadherin, vimentin, zo-1, and n-cadherin in wtCFTR- (**A**) and F508del (**B**)-expressing CFBE41o- cells following growth in different media. Expression was normalized to actin and expressed as fold change relative to MEM media. Similar relative trends were noted for immunoblot of e-cadherin and vimentin in wtCFTR- (**C**) and F508del (**D**)-expressing cells. Statistical comparisons were made within the protein of interest (e.g., vimentin:vimentin), comparing each media to MEM. OD was normalized to C4 Actin. Light grey—E-Cadherin; dark grey—vimentin. Using these immunoblot data, an e-cadherin to vimentin ratio was calculated for wtCFTR- (**E**) and F508del (**F**)-expressing cells. * *p* < 0.05; ** *p* < 0.01; *** *p* < 0.001; **** *p* < 0.0001 vs. MEM by one-way ANOVA with Dunnett’s multiple comparison test.

The relative expression of four commonly studied markers of epithelial and mesenchymal cell behavior were quantified by quantitative reverse transcriptase PCR. These included two epithelial markers, namely, e-cadherin, a component of the adherens junction, and zo-1, a component of the tight junction, as well as two mesenchymal markers, specifically, vimentin, an intermediate filament, and n-cadherin, a calcium-dependent transmembrane adhesion protein. For each gene, media-induced changes in expression were quantified as the fold-change compared to the MEM media. Across both F508del *CFTR*+ and wt*CFTR*+ cells, the expression of each gene of interest was generally highest in the MEM-treated cells (Figure 1A,B). The ALI-treated cells generally demonstrated the smallest reductions in epithelial gene expression (e-cadherin, zo-1), while the PC-treated cells demonstrated the smallest reduction in vimentin expression (Figure 1A,B). In addition, absolute expression, measured as the dCT values versus an actin control, was calculated for each gene of interest. For the wt*CFTR*+ cells, the expression was highest (lowest dCT) for e-cadherin, and lowest for zo-1 (average dCT values of ecad: 14.2, vim: 18.0, ncad: 19.7, and zo-1: 23.9; *p* < 0.0001 by one-way ANOVA). A similar pattern was noted in the F508del *CFTR*+ cells (average dCT values of ecad: 14.4, ncad: 18.5, vim: 19.5, and zo-1: 24.4; *p* < 0.0001 by one-way ANOVA). Between the wt*CFTR*+ and F508del *CFTR*+ cells, the basal expression levels of e-cadherin and zo-1 were equivalent (*p* = 0.94 and 0.61, respectively), while vimentin and n-cadherin were different (*p* = 0.002 and 0.009, respectively, by two-way ANOVA). 

The protein expression levels of e-cadherin and vimentin were then quantified using Western blot. A similar overall pattern of expression was noted in the cells treated with USG and ALI media, while the MEM-exposed cells also demonstrated increased e-cadherin compared to vimentin across both cell lines (Figure 1C,D). For the cells grown in PC media, similar expression and protein levels of e-cadherin and vimentin were observed in the wt*CFTR*+ cells, and there were higher levels of vimentin expression and protein in the F508del *CFTR*+ cells (Figure 1A–D). Consequently, when comparing the protein levels of e-cadherin:vimentin, the cells grown in PC displayed the most mesenchymal profile, which was more pronounced in the F508del *CFTR*+ cells. (Figure 1E,F). 

### 2.2. CFTR Expression

**Figure 2:**
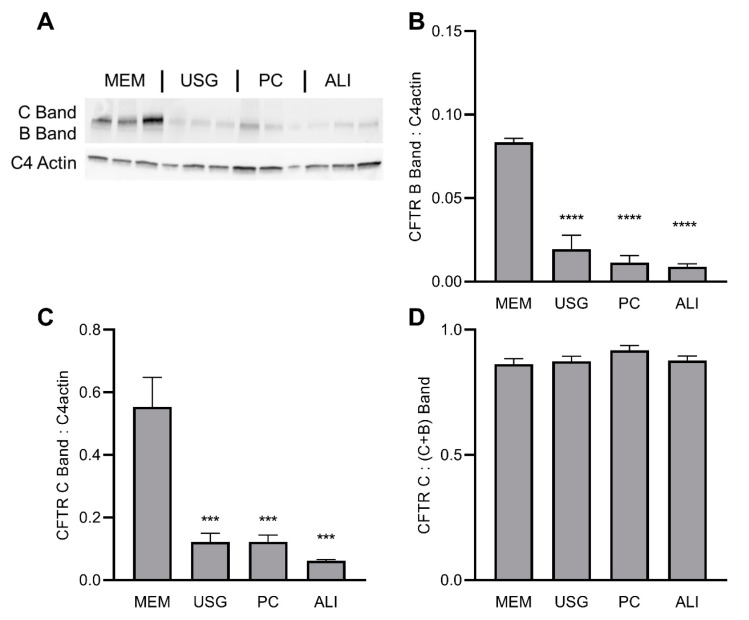
wtCFTR protein expression is increased in MEM media relative to USG, PC, and ALI media. Immunoblot for CFTR was performed and quantified for wtCFTR-expressing CFBE41o- cells (**A**). Densitometry was calculated relative to C4 actin and presented for Band B (immature) (**B**) and Band C (glycosylated) (**C**). The fraction of glycosylated wtCFTR protein (Band C/[Band C + Band B]) is quantified in (**D**). *** *p* < 0.001; **** *p* < 0.0001 vs. MEM by one-way ANOVA with Dunnett’s multiple comparisons correction.

**Figure 3:**
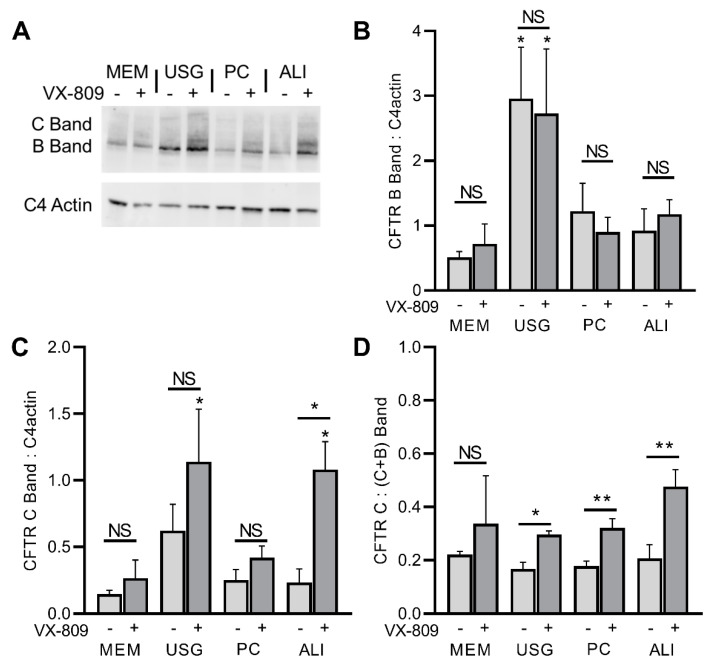
F508del CFTR protein expression is increased in USG media, with similar VX-809-induced rescue of C Band in all media. Immunoblot for CFTR was performed and quantified for untreated and VX-809-treated F508del CFTR-expressing CFBE41o- cells (**A**). Densitometry was calculated relative to C4 actin and presented for Band B (immature) (**B**) and Band C (glycosylated) (**C**). The fraction of glycosylated wtCFTR protein (Band C/[Band C + Band B]) is quantified in (**D**). Light grey bars—vehicle; dark grey bars—VX-809. For each within-media-vehicle- and VX-809-treated pair (e.g., MEM with/without VX-809), comparisons by 2-tailed T test are presented above the relevant paired bars. Comparisons across media within treatment categories (e.g., MEM, USG, PC, and ALI treated with vehicle) were conducted by one-way ANOVA with Dunnett’s multiple comparisons correction, noting significant differences against MEM via asterisk(s) above the individual data bar. * *p* < 0.05; ** *p* < 0.01; NS: Not Significant.

To evaluate the effect of differing growth media on CFTR expression, we examined the protein levels of the wt and F508del CFTR lines via Western blot. As before, cells of the same passage were grown on semi-permeable inserts in four media, lysed, and their proteins were isolated for Western blot. In the wt*CFTR*+ cells, the CFTR protein levels were the highest in the cells grown in MEM media, though the ratio of fully glycosylated C-band to immature B-band protein was the same regardless of the media (Figure 2). Conversely, in the F508del CFTR+ cells, the highest protein levels were noted in the cells grown in USG media (Figure 3). When treated for 72 h with VX-809 (lumacaftor) to improve F508del trafficking to the cell surface, no significant changes were seen in the immature B-band, while a trend towards increased C-band levels was noted (this was significant for the ALI media only; Figure 3A–C). When these data were expressed as a fraction of mature C-band relative to total C + B Bands, a similar improvement in the percentage of mature protein was seen across all groups, though this did not achieve significance in the MEM-treated cultures (Figure 3D). 

### 2.3. CFTR Function and Modulation

To assess if the changes in protein expression were reflected in measurable differences in CFTR function or modulation, cells of the same passage were again grown on semi-permeable inserts in four media for 10 days. The cells were then mounted in Ussing chambers and CFTR function was quantified as short-circuit current (Isc). 

**Figure 4:**
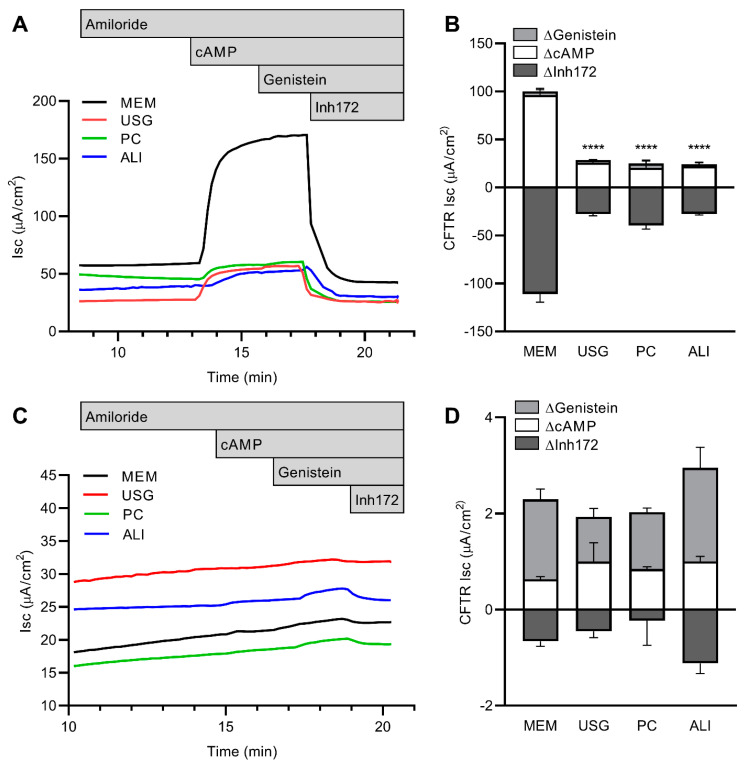
Media selection impacts wtCFTR but not F508del CFTR function in CFBE41o- cells. Cells grown in four media were mounted in Ussing chambers and CFTR function was quantified under voltage clamp conditions. In wtCFTR+ cells, significantly higher CFTR function was present in cells grown in MEM (representative Ussing tracings in (**A**); aggregate data in (**B**)), while this differential function was not noted in F508del CFTR+ cells (representative Ussing tracings in (**C**); aggregate data in (**D**)). White bars—ΔcAMP; light grey—ΔGenistein; dark grey—ΔInh172. n = 3–4 inserts per condition. **** *p* < 0.0001 vs. MEM by one-way ANOVA with Dunnett’s multiple comparisons correction.

In the wtCFTR+ cells, the stimulated CFTR function was significantly higher among the cells grown in MEM media, with reductions of >60% of function noted in the cells grown in USG, PC, or ALI media (Figure 4A,B). No appreciable amiloride-sensitive current was noted across any of the cultures, which is consistent with the previous characterization of this cell line. There were also no consistent differences in resistance or ATP-sensitive currents across any of the groups. 

**Figure 5:**
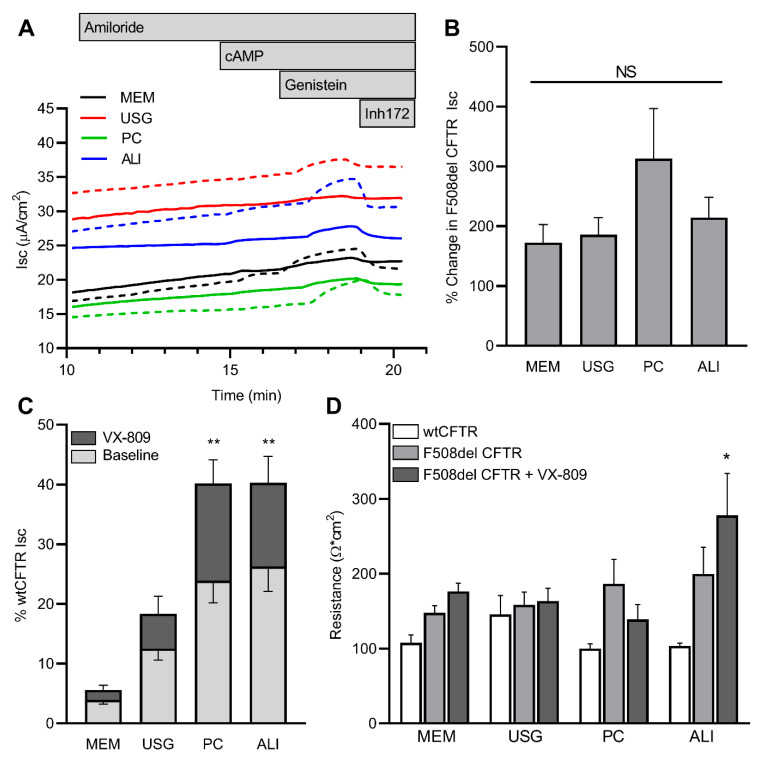
Media selection does not alter F508del CFTR rescue by VX-809; however, it does impact normalization of modulated CFTR function against wtCFTR. Cells grown in four media and treated for 72 h with vehicle or VX-809 were mounted in Ussing chambers and CFTR function was quantified under voltage clamp conditions. Representative tracings of corrected (dashed) and uncorrected (solid) F508del CFTR are shown in (**A**), while the VX-809-induced improvement in CFTR function is quantified in (**B**) as a relative change compared to vehicle. When normalized against media-specific wtCFTR function, there is a significant apparent difference in baseline (light grey) and VX-809-modulated (dark grey) F508del CFTR function (**C**); CFTR function is defined as cAMP- and genistein-dependent Isc, as presented in Figure 4B,D. Baseline resistance measurements for all conditions are shown in (**D**), with only VX-809-treated F508del inserts grown in ALI media being statistically different across the cohort. n = 3–4 inserts per condition. * p < 0.05; ** *p* < 0.01; NS: Not Significant vs. MEM by one-way ANOVA with Dunnett’s multiple comparison test (**B**,**C**), or two-way ANOVA with Tukey’s multiple comparisons test (**D**).

In the F508del CFTR+ cells, stimulated CFTR function was significantly reduced compared to the wtCFTR+ cells (Figure 4C,D). There was no significant difference in the stimulated CFTR function across the four media; however, there was a trend towards higher function in the cells grown in ALI media. To assess the degree of correction achieved with VX-809, a subset of cells was pretreated for 72 h. An increase in CFTR function over the baseline was seen across all four media types when the cells were treated with VX-809, with no significant difference in the relative increase over the baseline (Figure 5A,B). However, given the discrepancy in the media-dependent baseline function of the wtCFTR+ cells compared to the F508del CFTR+ cells (cAMP- and genistein-dependent changes in Isc, as seen in Figure 4B,D), when this pharmacologic rescue was quantified as the percentage of wild-type function, there appeared to be significantly higher baseline and rescued function in the cells grown in the PC and ALI media (Figure 5C). Baseline resistance was unchanged across all lines and treatment conditions, with the exception of the VX-809-treated F508del cells grown in ALI media (Figure 5D). 

## 3. Discussion

Herein, we report the effects of media selection on markers of epithelial and mesenchymal balance, as well as CFTR expression and activity in a stable human airway cell line. Cell lines are often studied as they are easy to grow and may provide more consistent results compared with primary cells obtained from different donors. As culture and model complexity grows, however, practices from primary cell culture such as media selection have been extrapolated to cell line-related work. These studies were conducted to understand the impact of media selection on the relevant epithelial and CFTR-specific nature of the CFBE41o- line in hopes of informing future comparisons of studies utilizing differing media. 

In this immortalized airway epithelial cell line, media selection altered the expression of relevant epithelial and mesenchymal markers. We chose e-cadherin (an epithelial marker) and vimentin (a mesenchymal marker), since they are both established markers, to monitor the epithelial:mesenchymal transition in prior publications [29,30]. While most media conditions produced increased e-cadherin expression relative to vimentin, suggesting an epithelial phenotype, growth in PC tended to enhance vimentin expression. This enhancement was further pronounced in the F508del CFTR-expressing cells (Figure 1). These results highlight the variability of expression in these markers produced by media selection, which may have a significant impact on cell behavior and experimental results.

The CFBE41o- cell line is frequently used in studies of CFTR expression and function, as the overexpression of this protein allows for convenient isolation and manipulation. As such, culture-dependent alterations in CFTR expression or function may have significant impacts on study findings and application. In this work, the wtCFTR+ cells demonstrated significantly higher levels of CFTR protein and function in the cells grown in MEM media compared to all others (Figure 2). Conversely, for the F508del CFTR+ cells, there was an increase in total CFTR protein in the cells grown in USG media (Figure 3). When treated with the CFTR modulator VX-809 (equivalent of lumacaftor), the cells grown in all four media demonstrated similar increases in mature CFTR protein (Figure 3). 

Functionally, the wtCFTR activity in the Ussing chambers mirrored this finding, with a significantly higher degree of wtCFTR function in the cells grown in MEM compared to other media (Figure 4). In the F508del CFTR+ cells, however, no difference was noted in baseline function across the media treatments (Figure 4). This discrepancy between protein and function in the F508del CFTR+ cells may represent the low overall level of function from this mutant protein. Importantly, the relative VX-809-induced functional rescue of F508del CFTR was similar across all media groups (Figure 5). Of note, however, due to the significant, media-dependent discrepancy in wtCFTR function without a similar change in F508del CFTR function, when the results regarding F508del are normalized to wtCFTR activity, there is a dramatic difference with respect to media type, with higher normalized baseline and rescued function in the cells grown in PC or ALI (Figure 5). Both the wild-type and F508del CFTR function (±VX-809) quantified in this study are similar to other published reports in this cell line, although the published ranges for both lines are wide [14,15,16]. 

The normalization of mutant CFTR functional studies to wtCFTR activity is a common practice, and the relative “percent of normal” CFTR activity has been utilized as a threshold for the regulatory expansion of clinical CFTR modulators [31]. The present data may be interpreted in several ways. One such interpretation is that the difference induced by media selection only occurs in the wtCFTR cells. This subsequently leads to the alteration of the denominator for the normalization of F508del CFTR function and the artificial creation of a difference in the F508del data. An alternate interpretation is that media selection also impacts F508del CFTR function, and that there is truly increased rescue based on growth media. The lack of difference in the baseline F508del CFTR function across the media groups provides evidence against this hypothesis. However, regardless of whether this difference is biological or mathematical, the observation that the use of different media may alter the relationship between wtCFTR+ and F508del CFTR+ data is critical for determining whether studies may be reliably compared. 

There are several key limitations to this study. First, only e-cadherin and vimentin were chosen to assess epithelial and mesenchymal states. These markers were chosen due to their frequent usage in airway epithelial studies. This approach was selected to demonstrate the potential differences in cellular phenotype caused by media selection. However, the epithelial and mesenchymal phenotypes are complex, and the distillation of this comparison to two genes constitutes a reductionist approach. Numerous additional epithelial and mesenchymal markers have been described, including through single-cell sequencing and phenotyping [32,33]. A more detailed characterization may lead to more comprehensive knowledge of this effect. Additional time in each culture media may also result in more phenotypic changes; however, the culture time must be balanced with the passage time in this rapidly growing cell line.

The comparisons of CFTR expression were made using Western blot only. CFTR mRNA levels were not quantified under the assumption of equivalent expression, as this construct is driven by a constitutively active CMV promoter. This approach, however, may not account for changes in mRNA stability. Additionally, the use of alternative anti-CFTR antibodies (other than UNC clone 570) may also result in slight differences in protein quantification. Nonetheless, the use of functional CFTR studies confirms the ultimate product of CFTR expression as it applies to cellular physiology. The relevance of VX-809 as a CFTR modulator for these studies is also notable. Though more effective modulator compounds exist in labs and clinics, specifically, elexacaftor combined with tezacaftor and ivacaftor, VX-809 was intentionally chosen for these studies to provide a low level of correction. In this way, VX-809 provides the minimal desired detectable level of correction for future work.

In summary, these studies in a stable human airway cell line demonstrated significant, media-dependent impacts on cell phenotype and CFTR expression and function, and these differences varied based on CFTR expression status. Attention to cell behavior in different growth conditions, including in stable cell lines, should be considered when examining airway cell biology and CFTR activity. In light of the above, we recommend the formulation of standardization between the various laboratories in order to allow for uniformity in the reporting of results. Based upon precedents and the robust functional rescue of CFTR seen in the “classical” MEM-based media, our group proposes that this approach should be utilized as a standard method of culture, with exceptions or changes made only when necessary and relevant to the desired outcomes and analyses.

## 4. Materials and Methods

Cell Culture. CFBE41o- cells were maintained longitudinally in MEM (Thermo Fisher Scientific, Waltham, MA, USA) with 10% FBS (Corning, Corning, NY, USA) and 1% penicillin/streptomycin (Quality Biological, Gaithersburg, MD, USA) in a humidified incubator at 37 °C and with 5% CO_2_ on fibronectin/collagen-coated dishes [2]. Cells were passaged onto 6.5 mm permeable inserts and exposed to different media for 7–10 days (apical and basolateral surfaces). To assess the impact of different media selection, cells were exposed to either MEM [25] or to three media traditionally used during the differentiation phase of primary cell culture: Ultroser-G^TM^-based media (USG) [26], a media specifically optimized for air–liquid interface culture (ALI media, purchased from https://www.med.unc.edu/mlicellcore/services/) [27], and a commercially available proprietary media (PneumaCult^TM^—ALI media) (PC media) [28]. VX-809 (Selleck Chemicals, Houston, TX, USA; 3 μM) was included for 72 h to correct F508del CFTR processing where applicable.

RNA Isolation and RT qPCR. Expression of epithelial (e-cadherin) and mesenchymal (vimentin) markers was monitored by RT qPCR as previously described [34,35]. In brief, cells on inserts were lysed and RNA was isolated using the commercially available RNeasy Mini Kit (Qiagen, Venlo, The Netherlands). Quality control was assured using a Nanodrop 2000c (Thermo Fisher Scientific). RNA expression of relevant genes was quantified via RT qPCR normalized to actin as a housekeeping gene and then expressed as fold-change compared to MEM.

Protein Isolation. Isolation and quantification of e-cadherin, vimentin, and CFTR was performed as previously described [36]. In brief, cells were lysed with Complete Lysis-M Buffer (Roche Diagnostics, Risch-Rotkreuz, Switzerland) and manually scraped with a pipette tip. The lysate was then centrifuged at 300× *g* for 5 min and the supernatant was separated from the cell pellet. Protein quantification was performed with the BioRad DC Protein Assay kit (Bio-Rad, Hercules, CA, USA) and immunoblot was performed using established Western blot techniques with densitometric quantification [37]. Antibodies used include e-cadherin (Rabbit mAb #3195, Cell Signaling Technology, Danvers, MA, USA), vimentin (Rabbit mAb #5741, Cell Signaling Technology), CFTR (mouse anti-CFTR clone 570, University of North Carolina at Chapel Hill, NC, USA), and C4 actin (mouse anti-Actin (C4), cat# LMAB-C4, Seven Hills Bioreagents, Cincinnati, OH, USA). 

CFTR Function. CFTR function was measured in Ussing chambers and studied as previously described [38]. Briefly, short-circuit current (Isc) and resistance were measured under voltage-clamp conditions using Acquire and Analyze 2.3 software. Cells were studied in an asymmetric chloride solution (6 mM Cl^−^ apical buffer) to produce a basolateral-to-apical Cl^−^-secretory gradient. Then, cells were treated apically with amiloride (sodium-channel blocker, 100 μM). CFTR was stimulated with forskolin (activator of transmembrane AC, 10 μM) and IBMX (nonspecific PDE-inhibitor, 100 μM) in both compartments. Genistein (50 μM) was added apically to potentiate CFTR. At the end of all studies, CFTR Inhibitor-172 (10 μM) was added to the apical compartment to block CFTR. 

Statistical analyses. All studies included 3–4 inserts per condition and were repeated in duplicate or triplicate with consistent results. All data were imported into Prism v9.3.1 (GraphPad Software, LLC, San Diego, CA, USA) for analysis. Unpaired, 2-tailed *t* tests (2 group comparisons, with Holm–Šidák correction for multiple comparisons where applicable) and one- or two-way ANOVA (multiple groups, with Dunnett’s or Tukey’s multiple comparison test where applicable) were used to compare continuous data, with an a priori α (*p*) value less than 0.05 used to determine statistical significance. Mean estimates (±SEM) are presented for comparison of continuous data. 

## 5. Conclusions

The present studies in a stable human airway cell line demonstrated significant, media-dependent impacts on cell behavior, and these differences varied based on CFTR expression status. Cell behavior under different growth conditions, including in stable cell lines, should be considered when examining airway cell biology and CFTR function. The generation of standardized media approaches and method reporting across laboratories utilizing such models will improve transparency and the ability to compare results across studies. 

## Data Availability

The data presented in this publication are available upon request from the corresponding author.

## References

[1] Rowe S.M., Miller S., Sorscher E.J. (2005). Cystic Fibrosis. N. Engl. J. Med..

[2] Welsh M.J. (1986). An apical-membrane chloride channel in human tracheal epithelium. Science.

[3] Gentzsch M., Boyles S.E., Cheluvaraju C., Chaudhry I.G., Quinney N.L., Cho C., Dang H., Liu X., Schlegel R., Randell S.H. (2017). Pharmacological rescue of conditionally reprogrammed cystic fibrosis bronchial epithelial cells. Am. J. Respir. Cell Mol. Biol..

[4] Gottschalk L.B., Vecchio-Pagan B., Sharma N., Han S.T., Franca A., Wohler E.S., Batista D.A.S., Goff L.A., Cutting G.R. (2016). Creation and characterization of an airway epithelial cell line for stable expression of CFTR variants. J. Cyst. Fibros..

[5] Gentzsch M., Mall M.A. (2018). Ion Channel Modulators in Cystic Fibrosis. Chest.

[6] Kunzelmann K., Schwiebert E.M., Zeitlin P.L., Kuo W.L., Stanton B.A., Gruenert D.C. (1993). An immortalized cystic fibrosis tracheal epithelial cell line homozygous for the delta F508 CFTR mutation. Am. J. Respir. Cell Mol. Biol..

[7] Ehrhardt C., Collnot E.-M., Baldes C., Becker U., Laue M., Kim K.-J., Lehr C.-M. (2006). Towards an in vitro model of cystic fibrosis small airway epithelium: Characterisation of the human bronchial epithelial cell line CFBE41o-. Cell Tissue Res..

[8] Swiatecka-Urban A., Brown A., Moreau-Marquis S., Renuka J., Coutermarsh B., Barnaby R., Karlson K.H., Flotte T.R., Fukuda M., Langford G.M. (2005). The short apical membrane half-life of rescued ΔF508-cystic fibrosis transmembrane conductance regulator (CFTR) results from accelerated endocytosis of ΔF508H-CFTR in polarized human airway epithelial cells. J. Biol. Chem..

[9] Varga K., Goldstein R.F., Jurkuvenaite A., Chen L., Matalon S., Sorscher E.J., Bebok Z., Collawn J.F. (2008). Enhanced cell-surface stability of rescued ΔF508 cystic fibrosis transmembrane conductance regulator (CFTR) by pharmacological chaperones. Biochem. J..

[10] Zhang D., Ciciriello F., Anjos S.M., Carissimo A., Liao J., Carlile G.W., Balghi H., Robert R., Luini A., Hanrahan J.W. (2012). Ouabain mimics low temperature rescue of f508del-CFTR in cystic fibrosis epithelial cells. Front. Pharmacol..

[11] Andersson C., Servetnyk Z., Roomans G.M. (2003). Activation of CFTR by genistein in human airway epithelial cell lines. Biochem. Biophys. Res. Commun..

[12] Hentchel-Franks K., Lozano D., Eubanks-Tarn V., Cobb B., Fan L., Oster R., Sorscher E., Clancy J.P. (2004). Activation of airway Cl- secretion in human subjects by adenosine. Am. J. Respir. Cell Mol. Biol..

[13] Sondo E., Tomati V., Caci E., Esposito A.I., Pfeffer U., Pedemonte N., Galietta L.J.V. (2011). Rescue of the mutant CFTR chloride channel by pharmacological correctors and low temperature analyzed by gene expression profiling. Am. J. Physiol.-Cell Physiol..

[14] Stanton B.A., Coutermarsh B., Barnaby R., Hogan D. (2015). Pseudomonas aeruginosa reduces VX-809 stimulated F508del-CFTR chloride secretion by airway epithelial cells. PLoS ONE.

[15] Farinha C.M., Sousa M., Canato S., Schmidt A., Uliyakina I., Amaral M.D. (2015). Increased efficacy of VX-809 in different cellular systems results from an early stabilization effect of F508del-CFTR. Pharmacol. Res. Perspect..

[16] Raraigh K.S., Paul K.C., Goralski J.L., Worthington E.N., Faino A.V., Sciortino S., Wang Y., Aksit M.A., Ling H., Osorio D.L. (2022). CFTR bearing variant p.Phe312del exhibits function inconsistent with phenotype and negligible response to ivacaftor. JCI Insight.

[17] Varelogianni G., Oliynyk I., Roomans G.M., Johannesson M. (2010). The effect of N -acetylcysteine on chloride efflux from airway epithelial cells. Cell Biol. Int..

[18] Carbone A., Zefferino R., Beccia E., Casavola V., Castellani S., Di Gioia S., Giannone V., Seia M., Angiolillo A., Colombo C. (2018). Gap Junctions Are Involved in the Rescue of CFTR-Dependent Chloride Efflux by Amniotic Mesenchymal Stem Cells in Coculture with Cystic Fibrosis CFBE41o-Cells. Stem Cells Int..

[19] Murgia X., Yasar H., Carvalho-Wodarz C., Loretz B., Gordon S., Schwarzkopf K., Schaefer U., Lehr C.-M. (2017). Modelling the bronchial barrier in pulmonary drug delivery: A human bronchial epithelial cell line supplemented with human tracheal mucus. Eur. J. Pharm. Biopharm..

[20] Okuda K., Dang H., Kobayashi Y., Carraro G., Nakano S., Chen G., Kato T., Asakura T., Gilmore R.C., Morton L.C. (2021). Secretory Cells Dominate Airway CFTR Expression and Function in Human Airway Superficial Epithelia. Am. J. Respir. Crit. Care Med..

[21] Hisert K.B., Heltshe S.L., Pope C., Jorth P., Wu X., Edwards R.M., Radey M., Accurso F.J., Wolter D.J., Cooke G. (2017). Restoring cystic fibrosis transmembrane conductance regulator function reduces airway bacteria and inflammation in people with cystic fibrosis and chronic lung infections. Am. J. Respir. Crit. Care Med..

[22] Neuberger T., Burton B., Clark H., Van Goor F. (2011). Use of primary cultures of human bronchial epithelial cells isolated from cystic fibrosis patients for the pre-clinical testing of CFTR modulators. Methods Mol. Biol..

[23] Awatade N.T., Wong S.L., Capraro A., Pandzic E., Slapetova I., Zhong L., Turgutoglu N., Fawcett L.K., Whan R.M., Jaffe A. (2021). Significant functional differences in differentiated Conditionally Reprogrammed (CRC)- and Feeder-free Dual SMAD inhibited-expanded human nasal epithelial cells. J. Cyst. Fibros..

[24] Lu S., Kolls J.K. (2022). Multi-omic comparisons between CFBE41o- cells stably expressing wild-type CFTR and F508del-mutant CFTR. J. Cyst. Fibros..

[25] Carbone A., Paracchini V., Castellani S., Gioia S., Seia M., Colombo C., Conese M. (2014). Human Amnion-Derived Cells: Prospects for the Treatment of Lung Diseases. Curr. Stem Cell Res. Ther..

[26] Voisin G., Bouvet G.F., Legendre P., Dagenais A., Massé C., Berthiaume Y. (2014). Oxidative stress modulates the expression of genes involved in cell survival in ΔF508 cystic fibrosis airway epithelial cells. Physiol. Genom..

[27] Fulcher M.L., Randell S.H. (2013). Human nasal and tracheo-bronchial respiratory epithelial cell culture. Methods Mol. Biol..

[28] Rayner R.E., Makena P., Prasad G.L., Cormet-Boyaka E. (2019). Optimization of Normal Human Bronchial Epithelial (NHBE) Cell 3D Cultures for in vitro Lung Model Studies. Sci. Rep..

[29] Kaszak I., Witkowska-Piłaszewicz O., Niewiadomska Z., Dworecka-Kaszak B., Toka F.N., Jurka P. (2020). Role of cadherins in cancer—A review. Int. J. Mol. Sci..

[30] Satelli A., Li S. (2011). Vimentin in cancer and its potential as a molecular target for cancer therapy. Cell. Mol. Life Sci..

[31] Van Goor F., Yu H., Burton B., Hoffman B.J. (2014). Effect of ivacaftor on CFTR forms with missense mutations associated with defects in protein processing or function. J. Cyst. Fibros..

[32] Danopoulos S., Bhattacharya S., Mariani T.J., Al Alam D. (2020). Transcriptional characterisation of human lung cells identifies novel mesenchymal lineage markers. Eur. Respir. J..

[33] Travaglini K.J., Nabhan A.N., Penland L., Sinha R., Gillich A., Sit R.V., Chang S., Conley S.D., Mori Y., Seita J. (2020). A molecular cell atlas of the human lung from single-cell RNA sequencing. Nature.

[34] Hudock K.M., Collins M.S., Imbrogno M., Snowball J., Kramer E.L., Brewington J.J., Gollomp K., McCarthy C., Ostmann A.J., Kopras E. (2020). Neutrophil extracellular traps activate IL-8 and IL-1 expression in human bronchial epithelia. Am. J. Physiol.-Lung Cell. Mol. Physiol..

[35] Brewington J.J., Backstrom J., Feldman A., Kramer E.L., Moncivaiz J.D., Ostmann A.J., Zhu X., Lu L.J., Clancy J.P. (2018). Chronic β2AR stimulation limits CFTR activation in human airway epithelia. JCI Insight.

[36] Sun H., Harris W.T., Kortyka S., Kotha K., Ostmann A.J., Rezayat A., Sridharan A., Sanders Y., Naren A.P., Clancy J.P. (2014). TGF-beta downregulation of distinct chloride channels in cystic fibrosis-affected epithelia. PLoS ONE.

[37] Taylor S.C., Berkelman T., Yadav G., Hammond M. (2013). A defined methodology for reliable quantification of western blot data. Mol. Biotechnol..

[38] Brewington J.J., Filbrandt E.T., LaRosa F.J., Ostmann A.J., Strecker L.M., Szczesniak R.D., Clancy J.P. (2018). Detection of CFTR function and modulation in primary human nasal cell spheroids. J. Cyst. Fibros..

